# Author Correction: Ptch2/Gas1 and Ptch1/Boc differentially regulate Hedgehog signalling in murine primordial germ cell migration

**DOI:** 10.1038/s41467-020-16304-7

**Published:** 2020-05-05

**Authors:** Yeonjoo Kim, Jiyoung Lee, Maisa Seppala, Martyn T. Cobourne, Soo-Hyun Kim

**Affiliations:** 1 Molecular and Clinical Sciences Research Institute, St. George’s, University of London, Cranmer Terrace, London, SW17 0RE UK; 2 Centre for Craniofacial and Regenerative Biology, King’s College London, Guy’s Hospital, London, SE1 9RT UK

**Keywords:** Developmental biology, Germline development, Stem-cell niche

Correction to: *Nature Communications* 10.1038/s41467-020-15897-3, published online 24 April 2020

The original version of this Article contained an error in the author affiliations.

Affiliation 2 incorrectly read Centre for Craniofacial and Regenerative Biology, King’s College London, Guy’s Hospital, London SE1 9RT, UK.

This has now been corrected in both the PDF and HTML versions of the Article.

