# Supplementary material for: Ptch2/Gas1 and Ptch1/Boc differentially regulate Hedgehog signalling in murine primordial germ cell migration
Source: Nat Commun. 2020 Apr 24;11:1994. doi: 10.1038/s41467-020-15897-3 (PMC7181751; doi:10.1038/s41467-020-15897-3)
Supplement: Supplementary file 1 — Supplementary Information [file 41467_2020_15897_MOESM1_ESM.pdf]

Ptch2/Gas1 and Ptch1/Boc differentially regulate Hedgehog signalling in murine primordial germ cell migration

Yeonjoo Kim[1], Jiyoung Lee[1], Maisa Seppala[2], Martyn T. Cobourne[2] and Soo-Hyun Kim[1]*

[1]Molecular and Clinical Sciences Research Institute, St. George's, University of London, Cranmer Terrace, London SW17 0RE and [2]Centre for Craniofacial and Regenerative Biology, King's College London, Guy's Hospital, London SE1 9RT, United Kingdom

*Correspondence to skim@sgul.ac.uk

SUPPLEMENTARY TABLES

Supplementary Table 1. Primers used for RT-PCR

Gene	Genebank	Tm (°C)	Primer sequences (5' to 3')	Product size
18s	NR_003278.3	60	F: GCAATTATTCCCCATGAACG<br>R: GGCCTCACTAAACCATCCAA	123bp
Dhh	NM_007857.5	56	F: GGGACCTCGTACCCAACTAC<br>R: CTTTGCAACGCTCTGTCATC	139bp
Ihh	NM_010544.3	60	F: TGGACTCATTGCCTCCCAGA<br>R: CAGCGACTTCCTCACCTTCCT	102bp
Shh	NM_009170.3	60	F: CAGCGACTTCCTCACCTTCCT<br>R: AGCGTCTCGATCACGTAGAAG	129bp
Boc	NM_172506.2	55	F: TTCATCCCCTTCTGCCTATG<br>R: ACCATTGTGTACTGGCACGA	187bp
Cdon	NM_021339.2	55	F: CCAGTGCGTTGCCAACAACAGC<br>R: TGGTACCCTGCAGCCAATGAAGC	148bp
Gas1	NM_008086.2	55	F: TCAACGACTGCGTGTGCGATGG<br>R: GGACCGTTGCTCGCATCTGG	100bp
GFP	-	55	F: CGACGGCAACTACAAGAC<br>R: TAGTTGTACTCCAGCTTGTGC	129bp
Gli1	NM_010296.2	57	F: CTATCCTCAGCCTCCCCATG<br>R: CCTCCCACAACAATTCCTGC	146bp
Gli2	NM_001081125.1	57	F: CAGTCCTGAGCTATCCCCAG<br>R: GAGGCTGCATGAGACCAAAG	117bp
Gli3	NM_000168.5	57	F: CTGCAGTGAGAGTGGACAGG<br>R: GTATCCAGTTGTGGGCTGCT	162bp
Ptch1	NM_000264.3	55	F: TGTTCCAGTTAATGACTCCC<br>R: ACACTCTGATGAACCACCTC	145bp
Ptch2	NM_001312903.1	55	F: TCCAAGTATCACTCTATGGGAAATC<br>R: TTCTCAATCATCCGCTCGAT	103bp
Stella	NM_139218.1	60	F: CTTTCCCAAGAGAAGGGTCC<br>R: TGCAGAGACATCTGAATGGC	149bp
Gapdh	NM_001289726.1	59	F: CGTCCCGTAGACAAAATGGT<br>R: GAGGTCAATGAAGGGGTCG	129bp

Supplementary Table 2. Guide RNAs used for CRISPR/Cas9

Gene Target	20bp Targeting Sequences before PAM
WDR11	CTTCGACCCCAAACAACATG
Gas1	CAGCTCAACCACACGCGCCG
Ptch1	TAATCTCGAGACCAACGTGG

Supplementary Table 3. Primers used for validation of gene targeting after CRISPR/Cas9

Primer name	Primer for gene expression
WDR11F	TAGGGGTATTGAATGGACAAGC
WDR11R	CCAAAGAAAATGCTCTGAGGAC
Gas1F	CGACTGCAGCTACGCCTAC
Gas1R	ACCTGCAGTGTTCGTCCTG
Ptch1F	TGGTTGTGGGTCTCCTCATATT
Ptch1R	TACAAGGAGGCTCTAGGTGCAT

SUPPLEMENTARY FIGURES

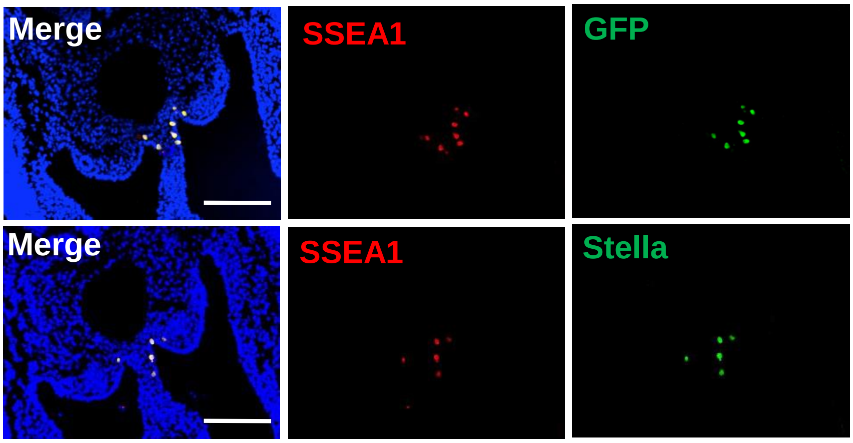

Supplementary Figure 1. Validation of Stella-GFP transgenic mouse embryos. Representative images of co-immunostaining on transverse sections of E10.5 embryos from Stella-GFP mouse using anti-SSEA1 and anti-GFP antibodies (top panel) and anti-SSEA1 and anti-Stella antibodies (bottom panel). n=5 independent biological samples. Scale bar, 100µm.

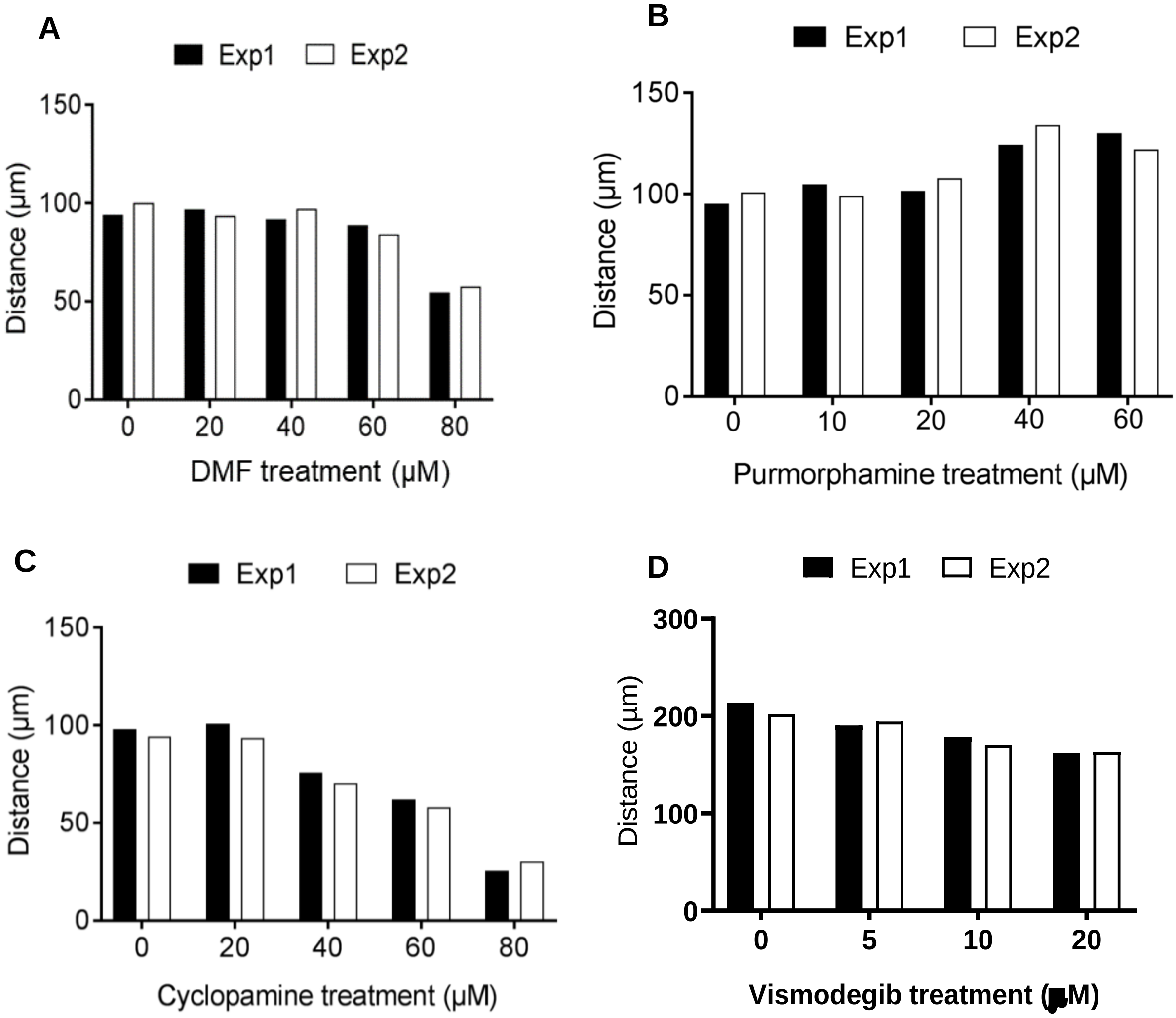

Supplementary Figure 2. Dose-dependent effects of Hh agonist and antagonist on PGC migration. Stella-GFP embryo slices at E10.5 were exposed to varying concentrations of the solvent DMF (A), purmorphamine (B) or cyclopamine (C). The graphs show the average migration distance of 7-10 PGCs in one embryo slice from two independent experiments imaged for 10 hours. The effects of Vismodegib (D) is assessed from the motility tracking of >20 PGCs in the E10.5 GR primary cultures imaged for 16 hours. Total accumulated distance is shown as an average.

Source data are provided as a Source Data file.

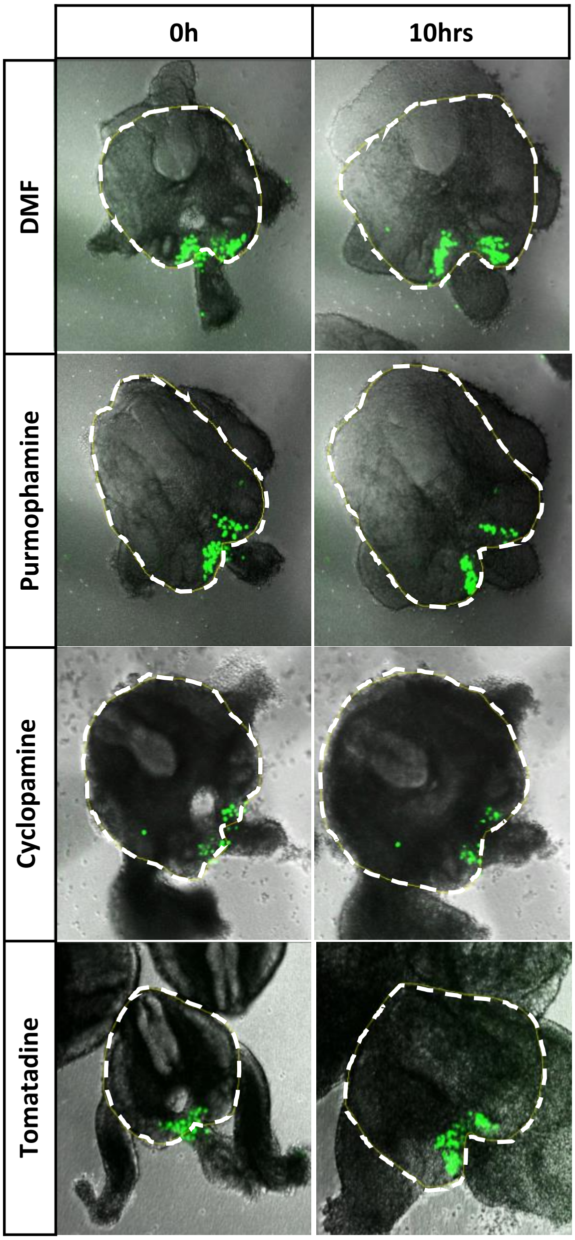

Supplementary Figure 3. The effects of Hh agonist and antagonist on embryo growth. Representative images of Stella-GFP embryo slices at E10.5 were exposed to varying concentrations of the solvent DMF (n=7), purmorphamine (n=7), cyclopamine (n=9) or tomatidine (n=6) in biologically independent experiments. The growth rate of embryos over the period of live imaging shown in the Supplementary Movies 1-4 was assessed by the changes of total area of embryo trunk (dotted line) in each embryo at the beginning (t = 0hr) and end (t = 10hr) of the movie.

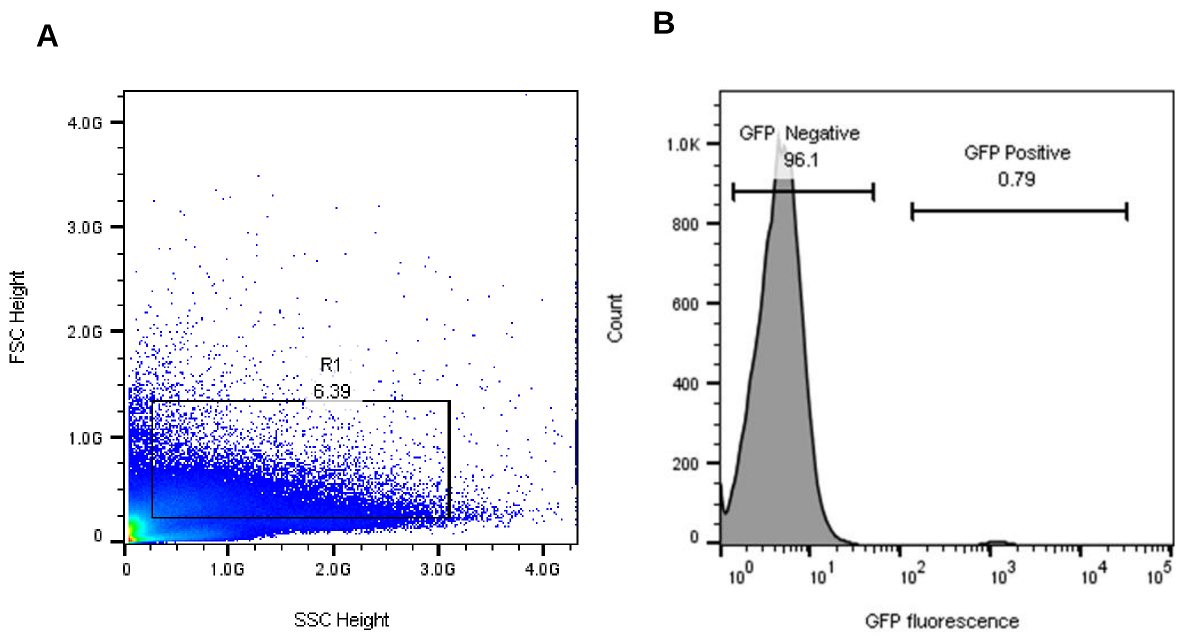

Supplementary Figure 4. Isolation of PGCs from primary cultures of E10.5 genital ridge cells derived from Stella-GFP mouse embryos.

(A) A representative scatter plot of cells isolated by FACS, plotted as side scatter (SSC) versus forward scatter (FSC). (B) Cells selected in R1 are then plotted according to their GFP fluorescence. Two gates were created: GFP-negative and GFP-positive. Cells collected as GFP-positive (approximately 0.08% of the total cell sorted) and an equal number of GFP-negative somatic cells (1,700 cells) were analysed. Representative data are shown from 3 biologically independent experiments

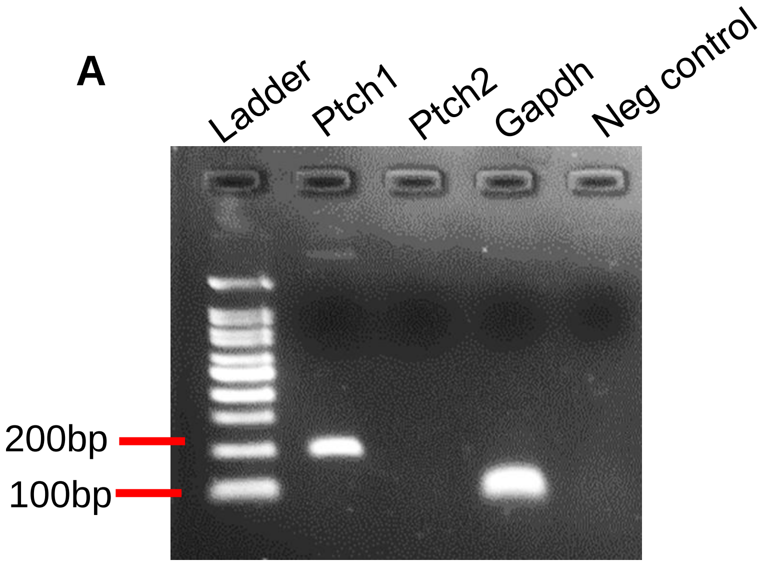

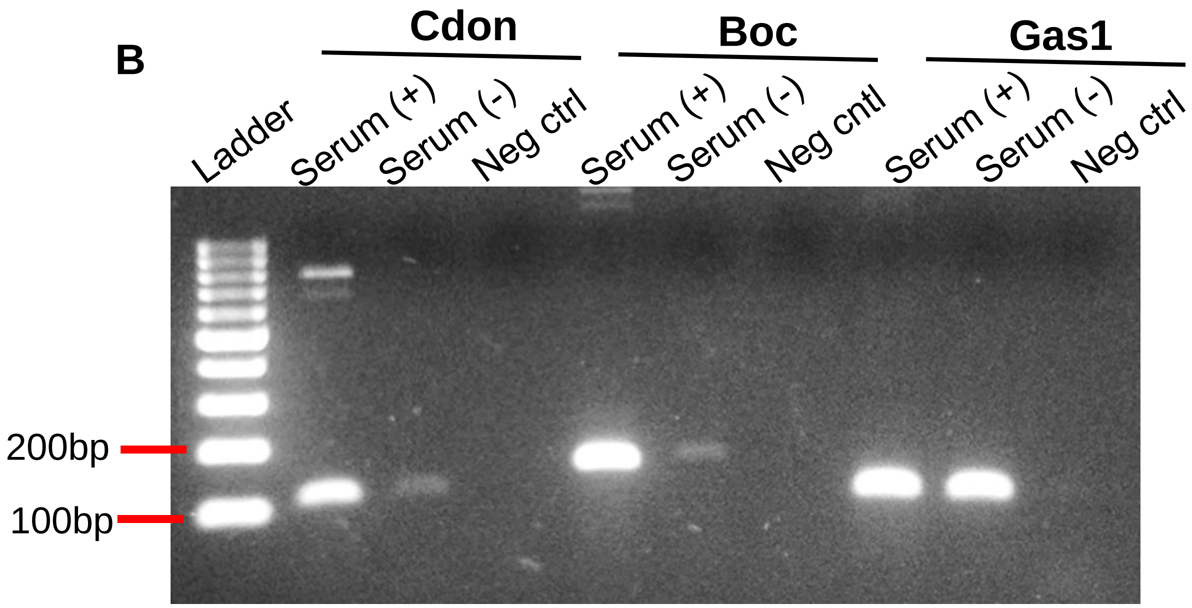

Supplementary Figure 5. Agarose gel images of RT-PCR analyses of Ptch1, Ptch2, Cdon, Boc and Gas1 in NIH3T3 cells.
(A) RT-PCR result of Ptch1 and Ptch2 with Gapdh as an internal control demonstrating that NIH3T3 cells do not express Ptch2 endogenously. (B) RT-PCR result of Cdon, Boc1 and Gas1 demonstrating that NIH3T3 cells express these receptors endogenously. Cultures with or without serum indicate some differences in the expression levels in Cdon and Boc. Representative images from 2 independent experiments are shown.

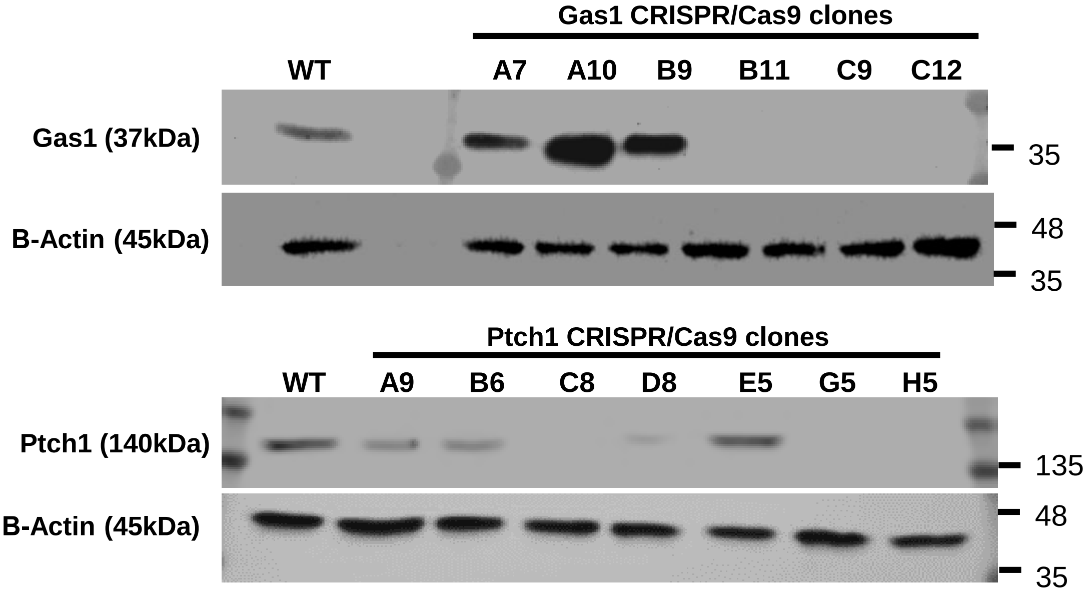

Supplementary Figure 6. Western blot analysis of NIH3T3 CRISPR/Cas9 clones after targeted gene knockout.
Different clones of NIH3T3 cells with targeted knockout of Gas1 and Ptch1 were analysed by Western blotting using specific antibodies as indicated. B-Actin as a loading control. After confirmation of the loss of endogenous protein expression, clone B11 from Gas1 KO and clone G5 from Ptch1 KO cells were used in further analyses. Representative blots are shown.
Source data are provided as a Source Data file.

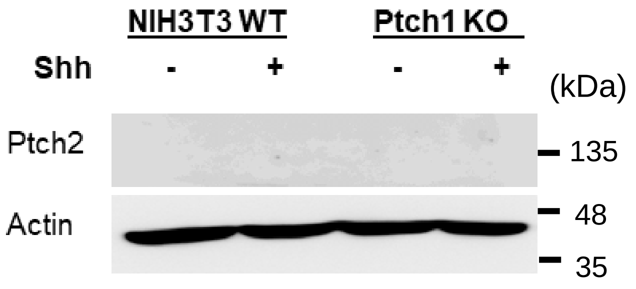

Supplementary Figure 7. Lack of Ptch2 expression in NIH3T3 cells.
Western blot analyses of NIH3T3 WT and Ptch1 KO cells with or without Shh ligand treatment for 40 minutes, confirming the lack of endogenous Ptch2 protein expression in NIH3T3, which is consistent with the RT-PCR results shown in Figure S5A. B-Actin as a loading control. Representative blots from 2 independent experiments are shown. Source data are provided as a Source Data file.

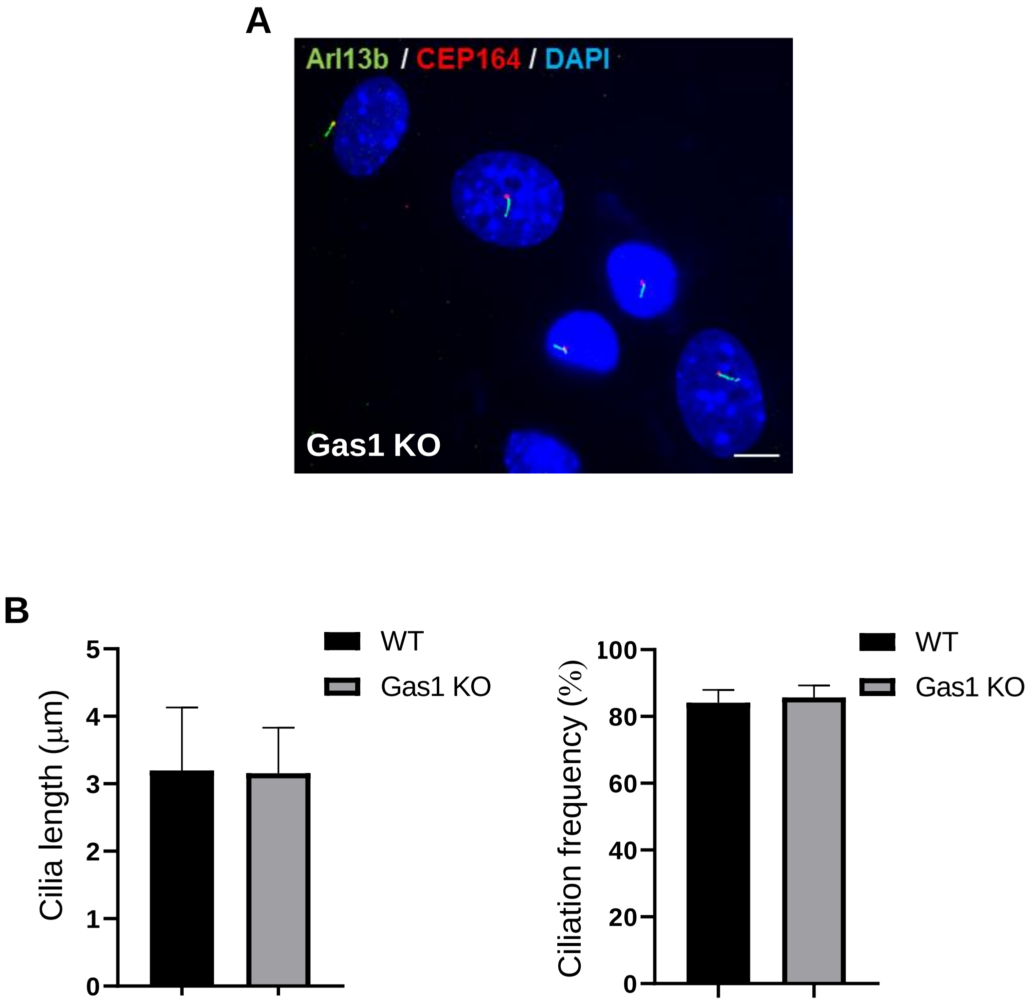

Supplementary Figure 8. Assessment of cilia status in Gas1 KO cells.
(A) Immunofluorescence staining of Gas1 KO NIH3T3/Cas9 cells using Arl13b and CEP164 antibody. Scale bar, 10µm. (B) The cilia length and frequency were compared between WT (n=186) and *Gas1* KO (n=120) cells. Statistical analyses by multiple t-test indicate no significant difference. Error bars represent SD.
Source data are provided as a Source Data file.
