# Supplementary material for: Ptch2/Gas1 and Ptch1/Boc differentially regulate Hedgehog signalling in murine primordial germ cell migration
Source: Nat Commun. 2020 Apr 24;11:1994. doi: 10.1038/s41467-020-15897-3 (PMC7181751; doi:10.1038/s41467-020-15897-3)
Supplement: Supplementary file 13 — Reporting Summary [file 41467_2020_15897_MOESM13_ESM.pdf]

nature research

Corresponding author(s): DBPR (NCOMMS-19-28270)

Last updated by author(s): Jan 16, 2020

Reporting Summary

Nature Research wishes to improve the reproducibility of the work that we publish. This form provides structure for consistency and transparency in reporting. For further information on Nature Research policies, see Authors & Referees and the Editorial Policy Checklist .

Statistics

For all statistical analyses, confirm that the following items are present in the figure legend, table legend, main text, or Methods section.

n/a	Confirmed	
☐	☒	The exact sample size (*n*) for each experimental group/condition, given as a discrete number and unit of measurement
☐	☒	A statement on whether measurements were taken from distinct samples or whether the same sample was measured repeatedly
☐	☒	The statistical test(s) used AND whether they are one- or two-sided<br>*Only common tests should be described solely by name; describe more complex techniques in the Methods section.*
☐	☒	A description of all covariates tested
☐	☒	A description of any assumptions or corrections, such as tests of normality and adjustment for multiple comparisons
☐	☒	A full description of the statistical parameters including central tendency (e.g. means) or other basic estimates (e.g. regression coefficient) AND variation (e.g. standard deviation) or associated estimates of uncertainty (e.g. confidence intervals)
☐	☒	For null hypothesis testing, the test statistic (e.g. *F*, *t*, *r*) with confidence intervals, effect sizes, degrees of freedom and *P* value noted<br>*Give P values as exact values whenever suitable.*
☒	☐	For Bayesian analysis, information on the choice of priors and Markov chain Monte Carlo settings
☒	☐	For hierarchical and complex designs, identification of the appropriate level for tests and full reporting of outcomes
☒	☐	Estimates of effect sizes (e.g. Cohen's *d*, Pearson's *r*), indicating how they were calculated

Our web collection on statistics for biologists contains articles on many of the points above.

Software and code

Policy information about availability of computer code

Data collection	Time-lapse image sequences from embryo slice culture and cell motility were analyzed by ImagePro Plus.<br>Live image cell tracking was performed using ImageJ plugin Chemotaxis and Migration tool (ibidi GmbH, Martinsried, Germany).<br>Quantitative real-time PCR data were collected by Light-Cycler 2.0 software (Roche).<br>Guide RNAs were designed by CRISPR Design Tool (http://crispr.mit.edu).<br>Fluorescence microscopy images were taken by Zeiss Axioplan 2 Upright microscope and analyzed using Fiji ImageJ software (National Institutes of Health).
Data analysis	Statistical analysis by GraphPad Prism 5 (La Jolla, CA, USA).

For manuscripts utilizing custom algorithms or software that are central to the research but not yet described in published literature, software must be made available to editors/reviewers. We strongly encourage code deposition in a community repository (e.g. GitHub). See the Nature Research guidelines for submitting code & software for further information.

Data

Policy information about availability of data

All manuscripts must include a data availability statement. This statement should provide the following information, where applicable:

- Accession codes, unique identifiers, or web links for publicly available datasets
- A list of figures that have associated raw data
- A description of any restrictions on data availability

The source data underlying Figures 2A, 2C, 2D, 3A-D, 4B, 4D, 5B, 5D, 6A, 6C, 6E, 6F, 7A, 7D, 7F, 7G and Supplementary Figures 2A-D, 3A, 7B are provided as a Source Data file. All relevant data are available from the authors.

Field-specific reporting

Please select the one below that is the best fit for your research. If you are not sure, read the appropriate sections before making your selection.

- [x] Life sciences
- [ ] Behavioural & social sciences
- [ ] Ecological, evolutionary & environmental sciences

For a reference copy of the document with all sections, see nature.com/documents/nr-reporting-summary-flat.pdf

Life sciences study design

All studies must disclose on these points even when the disclosure is negative.

Sample size	Using our experimental design, we expected to be able to detect 30-70% changes in those parameters which we considered to be biologically important in their effects. Based on the data from relevant publications and own experience with similar model systems, which provided approximate treatment effect sizes (standardized effect sizes of >3SD units) and standard deviations, we have calculated the sample sizes required to have 80% power to detect effects in the proposed experiments. Such sample sizes would have 80% power to detect standardized effect sizes of 1.2 SD units and larger at the P=0.05 significance level.
Data exclusions	Data exclusion criteria were pre-established. For mouse stufies, embryos that were not at the expected developmental stages after timed-mating or did not show the transgene expression or clear genotyping results were excluded. For in vitro assays, major outliers (a value that falls outside the data set's outer fences) that can be attributed to mistakes in measurement, recording or human errors are omitted.
Replication	All our experimental findings were based on the data obtained from multiple technical replicates as well as biological replicates, which were reproducible in independent experiments. The effects of subjective bias were minimised by blinding and randomisation.
Randomization	Samples were randomly assigned for studies using computerized random order generator (GraphPad, QuickCalcs).
Blinding	Researchers were blinded to genotypes or treatments while performing mouse tissue collection or in vitro assay analyses. Our breeding scheme generated litters with all experimental genotypes represented.

Reporting for specific materials, systems and methods

We require information from authors about some types of materials, experimental systems and methods used in many studies. Here, indicate whether each material, system or method listed is relevant to your study. If you are not sure if a list item applies to your research, read the appropriate section before selecting a response.

Materials & experimental systems

n/a	Involved in the study	
☐	☒	Antibodies
☐	☒	Eukaryotic cell lines
☒	☐	Palaeontology
☐	☒	Animals and other organisms
☒	☐	Human research participants
☒	☐	Clinical data

Methods

n/a	Involved in the study	
☒	☐	ChIP-seq
☐	☒	Flow cytometry
☒	☐	MRI-based neuroimaging

Antibodies

Antibodies used	Myc (M4439, mouse monoclonal 1:200, Sigma-Aldrich), Ptch1 (sc-293416, mouse monoclonal 1:200, Santa Cruz), Ptch2 (PA1-46223, rabbit polyclonal 1:200, Invitrogen), Gas1 (AF2636, goat polyclonal 1:500, R&D; PA5-48298, rabbit polyclonal 1:500), Boc (AF2385, goat polyclonal 1:500, R&D; MAB20361, mouse monoclonal 1:500, R&D), Smo (sc-166685, mouse monoclonal 1:200, Santa Cruz), Shh (sc-365112, mouse monoclonal 1:200, Santa Cruz), GLI3 (AF3690, goat polyclonal 1:200, R&D), WDR11 (ab175256, rabbit polyclonal 1:200, Abcam), SSEA1(MC-480, mouse monoclonal 1:200, DSHB), Stella (ab19878, rabbit polyclonal 1:200, Abcam), phospho-Src, (sc-166860, mouse monoclonal 1:500, Santa Cruz), phospho-Creb (sc-81486, mouse monoclonal 1:500, Santa Cruz), Arl13b (17711-1-AP, rabbit polyclonal 1:500, Proteintech), IFT88 (13967-1-AP, rabbit polyclonal 1:500, Proteintech), CEP164 (sc-515403, mouse monoclonal 1:200, Santa cruz), gamma□tubulin (T6557, mouse monoclonal 1:500, Sigma), b-actin (4967L, rabbit polyclonal 1:500, CST).
Validation	All commercial antibodies were initially validated by the providers as indicated in the manufacturer's website or product information sheets including citations. Some of the antibodies were further validated by us using positive and negative controls (e.g. cells transfected with an overexpression construct or targeted knockout samples) as described in the manuscript.

Eukaryotic cell lines

Policy information about cell lines

Cell line source(s)	HEK293 and NIH3T3 were purchased from American Type Culture Collection.
Authentication	The cell lines were authenticated by isoenzyme (interspecies) and STR (intraspecies) analysis by ATCC.
Mycoplasma contamination	All cell lines were negative for mycoplasma contamination.
Commonly misidentified lines (See ICLAC register)	N/A

Animals and other organisms

Policy information about studies involving animals; ARRIVE guidelines recommended for reporting animal research

Laboratory animals	Mouse strains C57BL6 and CD1/C57BL6 background. Both sexes were used for mating (adult 8 weeks - 5 months). Pregnant females were used for collecting embryos at embryonic day 9.5 - 11.5.
Wild animals	The study did not involve wild animals.
Field-collected samples	The study did not involve samples collected from the field.
Ethics oversight	All studies were conducted in accordance of Animal [Scientific Procedures] Act 1986 implemented by the Home Office in England. All mice were housed at the animal facilities of the authors' institutes under the approved institutional protocols and local regulations following the national guidelines for the care and use of laboratory animals.

Note that full information on the approval of the study protocol must also be provided in the manuscript.

Flow Cytometry

Plots

Confirm that:

- [x] The axis labels state the marker and fluorochrome used (e.g. CD4-FITC).
- [x] The axis scales are clearly visible. Include numbers along axes only for bottom left plot of group (a 'group' is an analysis of identical markers).
- [x] All plots are contour plots with outliers or pseudocolor plots.
- [x] A numerical value for number of cells or percentage (with statistics) is provided.

Methodology

Sample preparation	Genital ridge tissues dissected from E10.5 Stella-GFP mouse embryos were digested in 0.25% trypsin, passed through a 0.4µm cell strainer and suspended in DMEM/L-15 medium supplemented with 20% knockout serum replacement (Invitrogen), 2mM L-glutamine, 0.1mM non-essential amino acids and 0.1mM 2-mercaptoethanol (Sigma-Aldrich), and resuspended in the sorting buffer (1mM EDTA, 25mM HEPES at pH7.0, 1% FBS).
Instrument	GFP+ and GFP- cell populations were separated using a MoFlo XDP high-speed cell sorter (Beckman Coulter).
Software	FlowJo
Cell population abundance	On average 0.5 million cells were analysed during sorting, and about 100,000 negative cells and 1,000 positive cells were sorted. GFP-positive populations represent approximately 0.1% of the total sorted sample.
Gating strategy	Positive cells had an intensity of >2 on log scale of GFP detection using 488nm laser for excitation and a filter (529/28 nm) for detection. Cells were selected using FSC (height) versus SSC (height) and a gate was prepared to eliminate debris or abnormally shaped cells.

- [x] Tick this box to confirm that a figure exemplifying the gating strategy is provided in the Supplementary Information.
