# Supplementary material for: Ptch2/Gas1 and Ptch1/Boc differentially regulate Hedgehog signalling in murine primordial germ cell migration
Source: Nat Commun. 2020 Apr 24;11:1994. doi: 10.1038/s41467-020-15897-3 (PMC7181751; doi:10.1038/s41467-020-15897-3)
Supplement: Supplementary file 14 — Description of Additional Supplementary Files [file 41467_2020_15897_MOESM14_ESM.pdf]

Title: Supplementary Movie 1.
Description: PGC migration in E10.5 embryo with the addition of DMF solvent control. A time-lapse movie of an embryo slice culture from E10.5 Stella-GFP+/+ in the presence of 40µM DMF.

Title: Supplementary Movie 2.
Description: PGC migration in E10.5 embryo with the addition of purmorphamine. A time-lapse movie of an embryo slice culture from E10.5 Stella-GFP+/+ in the presence of 40µM purmorphamine.

Title: Supplementary Movie 3.
Description: PGC migration in E10.5 embryo with the addition of cyclopamine. A time-lapse movie of an embryo slice culture from E10.5 Stella-GFP+/+ in the presence of 40µM cyclopamine.

Title: Supplementary Movie 4.
Description: PGC migration in E10.5 embryo with the addition of tomatidine. A time-lapse movie of an embryo slice culture from E10.5 Stella-GFP+/+ in the presence of 40µM tomatidine.

Title: Supplementary Movie 5.
Description: Motility of PGCs in primary GR cultures with the addition of DMF solvent control. A time-lapse movie of E10.5 Stella-GFP+/+ mouse GR cultures in the presence of 40µM DMF.

Title: Supplementary Movie 6.
Description: Motility of PGCs in primary GR cultures with the addition of purmorphamine. A time-lapse movie of E10.5 Stella-GFP+/+ mouse GR cultures in the presence of 40µM purmorphamine.

Title: Supplementary Movie 7.
Description: Motility of PGCs in primary GR cultures with the addition of Shh-N. A time-lapse movie of E10.5 Stella-GFP+/+ mouse GR cultures in the presence of 40µM Shh-N.

Title: Supplementary Movie 8.
Description: Motility of PGCs in primary GR cultures with the addition of cyclopamine. A time-lapse movie of E10.5 Stella-GFP+/+ mouse GR cultures in the presence of 40µM cyclopamine.

Title: Supplementary Movie 9.
Description: Motility of PGCs in primary GR cultures with the addition of vismodegib. A time-lapse movie of E10.5 Stella-GFP+/+ mouse GR cultures in the presence of 10µM Vismodegib.

Title: Supplementary Movie 10.
Description: Motility of PGCs in primary GR cultures with the addition of tomatidine. A time-lapse movie of E10.5 Stella-GFP+/+ mouse GR cultures in the presence of 40µM tomatidine.
